# New Insight Into the Structure-Activity Relationship of Sweet-Tasting Proteins: Protein Sector and Its Role for Sweet Properties

**DOI:** 10.3389/fnut.2021.691368

**Published:** 2021-06-18

**Authors:** Xiangzhong Zhao, Congrui Wang, Yue Zheng, Bo Liu

**Affiliations:** ^1^School of Food Science and Engineering, Qilu University of Technology (Shandong Academy of Sciences), Jinan, China; ^2^Shandong Aojing Biotechnology Co., Ltd., Zoucheng, China

**Keywords:** sweet-tasting proteins, intramolecular interaction forces, protein sector, network, sweetness, stability

## Abstract

Sweet-tasting protein is a kind of biomacromolecule that has remarkable sweetening power and is regarded as the promising sugar replacer in the future. Some sweet-tasting proteins has been used in foods and beverages. However, the structure and function relationship of these proteins is still elusive, and guidelines for their protein engineering is limited. It is well-known that the sweet-tasting proteins bind to and activate the sweet taste receptor T1R2/T1R3, thus eliciting their sweetness. The “wedge-model” for describing the interaction between sweet-tasting proteins and sweet taste receptor to elucidate their sweetness has been reported. In this perspective article, we revealed that the intramolecular interaction forces in sweet-tasting proteins is directly correlated to their properties (sweetness and stability). This intramolecular interaction pattern, named as “protein sector,” refers to a small subset of residues forming physically connections, which cooperatively affect the function of the proteins. Based on the analysis of previous experimental data, we suggest that “protein sector” of sweet-tasting proteins is pivotal for their sweet properties, which are meaningful guidelines for the future protein engineering.

## Introduction

Sweet-tasting proteins are originated from the natural plants and exhibit extraordinary sweetening power, which are regarded as suitable replacers of sugars and artificial sweeteners in the future to improve the health of human beings, such as the control of obesity, diabetes, and hyperlipemia (1). Eight sweet proteins have been characterized so far (miraculin, monellin, thaumatin, mabinlin, pentadin, curculin, brazzein, and neoculin), with three proteins monellin, brazzein, and thaumatin being well-studied. However, some sweet-tasting proteins have intrinsic shortcomings (e.g., low sweetness or thermostability) that limit their extensive applications. In recent years, protein engineering of sweet-tasting proteins to improve their performance has drawn more and more attention of researchers and entrepreneurs, and many variants of these proteins with modified properties have been constructed (2, 3).

## Structural Features of Sweet-Tasting Proteins

**Figure 1:**
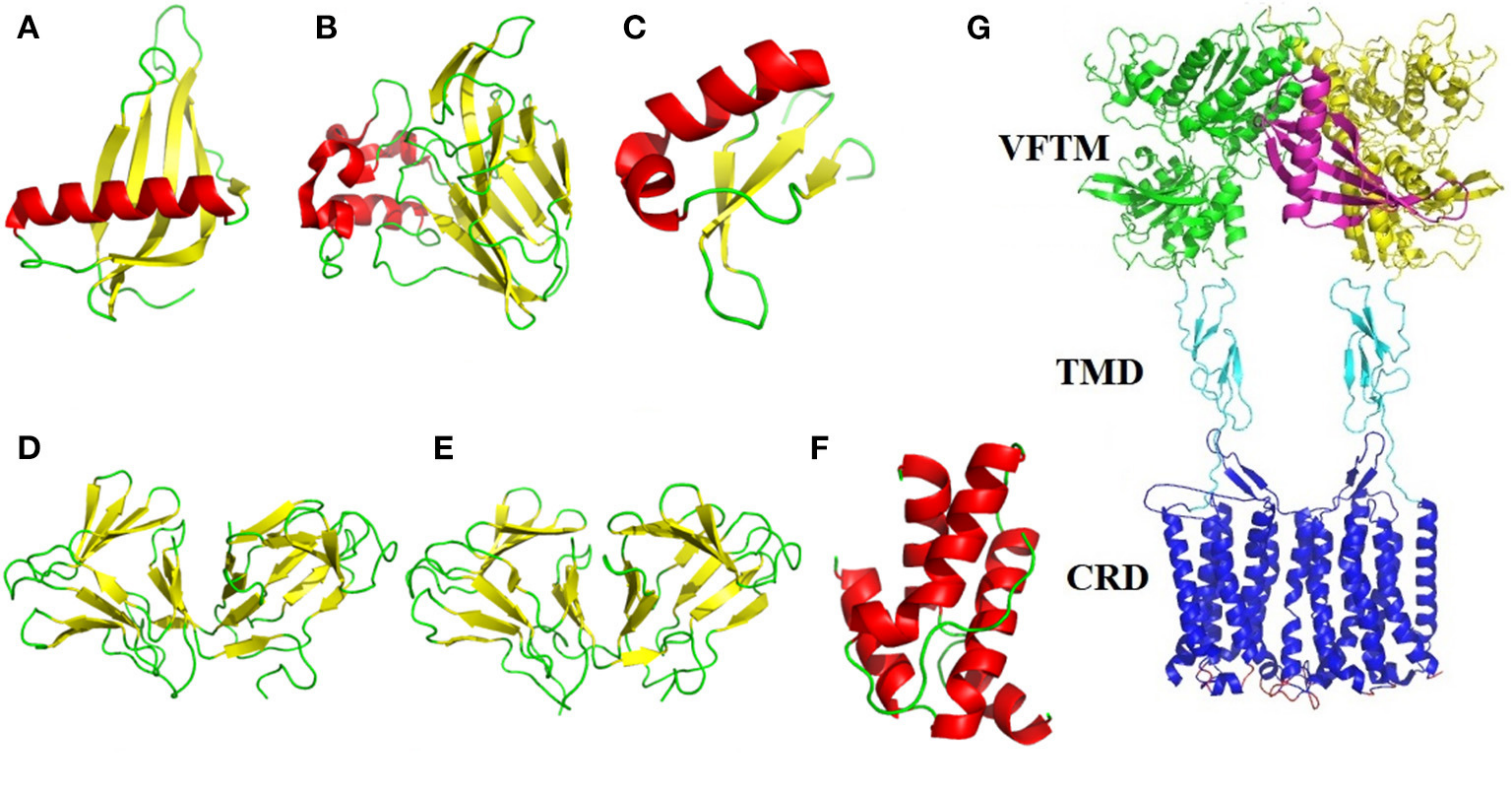
Structural illustration of sweet-tasting proteins and the sweet taste receptor. The three-dimensional structures of sweet-tasting proteins: **(A)** monellin (PDB: 2O9U); **(B)** thaumatin (PDB: 1RQW); **(C)** brazzein (PDB: 2LY5); **(D)** curculin (PDB: 2DPF); **(E)** neoculin (PDB: 2D04); **(F)** mabinlin II (PDB: 2DS2). The α-helix, β-sheet and loops in the structures were colored in red, yellow, and green, respectively. **(G)** Cartoon representation of the interaction between the sweet-tasting protein monellin (colored in purple) and the sweet taste receptor T1R2/T1R3. The VFTM (Venus flytrap module), CRD (cysteine-rich domain) and TMD (transmembrane domain) of the receptor are colored in green (T1R2) and yellow (T1R3), cyan and blue, respectively.

The sweet-tasting proteins consist of about 50–200 amino acids, with approximate molecular weight range from 6,500 to 30,000 Da. Interestingly, although these proteins display same properties (eliciting sweet sensation), they have no sequence identity and structural similarity. Indeed, the dimensional structures of many sweet-tasting proteins have been solved with X-ray diffraction or nuclear magnetic resonance (NMR), which show diversified spatial folding architectures including α-helix, β-sheet and random coils (loops) (Figures 1A–F).

## Monellin

Monellin is a sweet-tasting protein (molecular weight MW: 13,000 Da) about 3,000 times sweeter than sucrose on a weight basis, which was originally isolated from the fruit of the West African plant *Dioscoreophyllum cumminsii* Diels. The protein consists of two non-covalently associated polypeptide chains: an A chain of 44 amino acid residues and a B chain of 50 residues (4). A single-chain monellin (MNEI) was created by protein engineering in which the two natural chains are joined via a Gly-Phe dipeptide linker to improve its thermal stability (5). Interestingly, the spatial structures of native and single-chain monellin are very similar, which identically consist of a five-strand β-sheet partially “wrapped” around an α-helix (PDB: 4MON and 2O9U). Although the overall structure of monellin displays lower flexibility, the loops region in these structures show a high degree disorder, providing the structural plasticity that enables the protein to interact and optimize its large surface complementarity with the sweet taste receptor (6).

## Brazzein

Brazzein is the smallest, heat-stable and intensely sweet protein (MW: 6,473 Da) derived from the ripe fruit of the West African plant *Pentadiplandra brazzeana* Baillon, which consists of 54 amino acid residues (7). The structure of brazzein was determined by NMR (PDB ID: 1BRZ and 2BRZ), which shows one short α-helix (residues 21–29) and three strands of antiparallel β-sheet held together by four disulfide bonds. The protein adopts a cysteine-stabilized αβ (CSαβ) fold stabilized by 17 interstrand α-helical hydrogen bonds and four disulfide bridges (8). The structure of brazzein (PDB: 4HE7) solved by X-ray diffraction is alike to its overall solution structure solved by NMR but with essential difference (2.0-2.2 Å rmsd for the Cα atoms) found in the loop and terminal regions (9). Furthermore, the brazzein fold exhibits similarity to a family of serine proteinase inhibitors based on the sequence comparisons, suggesting that brazzein could evolved from a serine proteinase inhibitor through a deletion mutation (8).

## Thaumatin

Thaumatin is a 207 amino acids sweet-tasting protein (MW: 22,200 Da) with two major forms (I and II) and three minor forms (a, b, c), which is naturally derived from the fruit arils of a tropically grown plant *Thaumatococcus daniellii* Benth belonging to the family Marantaceae (10). The overall crystal structures of both natural and recombinant thaumatin I (PDB: 3AL7 and 2VHK) display three domains: a 11-strand flattened β-sandwich, a large disulfide-rich region and a small disulfide-rich region. The intramolecular eight disulfide bonds formed by sixteen cysteines are responsible for the stability of the protein (11). The quality of the 1.1 Å resolution of recombinant thaumatin I allowed the side chains of 20 residues to be modeled in two conformations and that of one residue (R76) to be modeled in three conformations. Study on the crystal structures of thaumation at different pHs has revealed that the increase in mobility of lysine residues as well as a loop region in domain II account for the pH-dependent sweetness of this sweet-tasting protein (12). Moreover, thaumatin has been approved and applied as both a sweetener and a favor-enhancer in many countries.

## Mabinlin

Mabinlin is isolated from the seeds of *Capparis masaikai* Levl, which grows in subtropical regions within China. Based on its sequence, this sweet-tasting protein can be categorized into four members I, II, III, and IV (13). The mabinlin II (MW: 12,400 Da) consists of an A-chain of 33 amino acids and a B-chain of 72 amino acids, which are linked through two interchain disulfide bridges. The crystal structure of mabinlin II (PDB: 2DS2) was determined in 2008, which belongs to the “all alpha protein” in SCOP (Structural Classification of Proteins) classification. Specifically, the A-chain has two α-helixs and B-chain has three α-helixs (no β-sheet), and four disulfide bridges exist in the protein molecule (14). Interestingly, the separated B-chain can elicit the sweetness, whereas the A-chain can not, which is in accordance to the further findings that the B-chain with a unique (NL/I) tetralet motif is the sweetness determinant site, while the A-chain may play a role for the long aftertaste of this sweet-tasting protein.

## Miraculin

Miraculin is a homodimeric sweet taste-modifying protein (191 amino acids, MW: 24,600 Da) which is isolated from the red berries of *Richadella dulcifera*, a shrub native to West Africa. Miraculin shows high amino acid sequence homology with the soybean trypsin inhibitor. The protein has no obvious taste at neutral pH. However, it has taste-modifying activity to convert sour stimuli to sweetness (15). Using human/mouse chimeric sweet taste receptors and molecular simulations, it has been revealed that miraculin binds with the human T1R2/T1R3 as an antagonist at neutral pH but functionally changes into an agonist at acidic pH, thus eliciting its sweet taste-modifying activity (16). Although the structure of miraculin is still not available, several crystal structures of miraculin-like proteins have been resolved, which show as a β-trefoil fold (PDB: 5YH4, IIR, etc.) (17).

## Curculin and Neoculin

Curculin is isolated from *Curculigo latifolia*, a plant grown in Malaysia. The homodimeric form of this protein (114 amino acids, MW: 14,600 Da) exhibits sweet taste-modifying activity as miraculin (18). However, its heterodimeric isoform (named as neoculin) exhibits both sweet-tasting and taste-modifying activities (19). Both crystal structures of curculin (PDB: 2DPF) and neoculin (PDB: 2D04) have been determined, which adopt very similar backbone conformation and domain arrangement (20). It is revealed that curculin exhibits sweetness and taste-modifying activities through its partially overlapping but distinct molecular surfaces, which are suggested to be involved in the interaction with the sweet taste receptor.

## Pentadin

Pentadin (500 times sweeter than sucrose on a weight basis, MW: 12,000 Da) has the same plant origin as brazzein (*Pentadiplandra brazzeana* Baillon), and its subunits are linked by an intramolecular disulfide bridge (21). The sweet properties of pentadin are more like that of monellin than that of thaumatin. However, there is no sequence and structural information reported about this sweet-tasting protein until now.

## Eliciting the Sweetness: Interaction Between the Sweet-Tasting Proteins and Sweet Taste Receptor

How the sweet-tasting proteins elicit their sweetness has been an intriguing question for a long time. In 2001–2002, scientists revealed that the heterodimeric receptor T1R2/T1R3 located in the membrane of taste bud cells mediates the sweet taste sensation upon the stimulus of various sweeteners (22, 23). The sweeteners (including sweet-tasting proteins) bind to, interact with, activate the receptor, then trigger a series of signal cascades (G protein activation, phospholipase C-β2 motivation, Ca^2+^ release, cell depolarization, etc.), and ultimately elicit the sweet sensation (24). However, structural determination of this membrane protein G protein-coupled receptor (GPCR) is still a big challenge, and spatial information of sweetener-receptor complex is lacking now. Nevertheless, molecular modeling and docking have been extensively performed to investigate the sweetener-receptor interaction, which could be verified by further functional mutagenesis analysis(25–27).

The most popular model elucidating the interaction between sweet-tasting proteins and receptor is called as wedge-model, proposed by prof. Temussi, in which the surface charge complementarity between the sweet-tasting proteins (or its amino acids) and the sweet taste receptor mediates their interaction thus determining the sweetness of proteins (Figure 1G) (28). This model has been broadly accepted according to molecular simulation and experimental validation (29, 30).

## Strategies for Optimization of the Properties of Sweet-Tasting Proteins

Guidance for protein engineering of the sweet-tasting proteins is primarily based on the above described wedge-model. Accordingly, mutated residues of the sweet-tasting protein variants were mainly focused on those on their protein surface, and removing negative charge or increasing positive charge is generally accompanied with improved sweetness, which is consistent with their charge complementarity with the interactive residues (negative charge) in sweet taste receptor. For instance, mutants E2N and Y65R of MNEI, E41K, D40K, and E53R of brazzein, and D21N of thaumatin with significantly improved sweetness have been constructed (31–35). Notably, structural calculations and quantitative structure-activity relationship (QSAR) investigations have been popularly performed in recent years to improve the properties of sweet-tasting proteins with some novel mutants having been identified (e.g., S76Y of MNEI) (36, 37).

## “Proteins Sectors” is Correlated to the Sweetness of Sweet-Tasting Proteins

In 2009, Halabi et al. uncovered that biological properties of proteins arise from the cooperative action of their amino acid residues, and the pattern of residue cooperativity is generally called as “protein sector,” in which a small subset of residues forms an interactive architecture and physically connected networks, and each sector is physically connected in the tertiary structure and has a distinct functional role (38, 39). In recent years, other interdisciplinary methods and techniques have been adopted to identify the protein sectors (40, 41).

**Figure 2:**
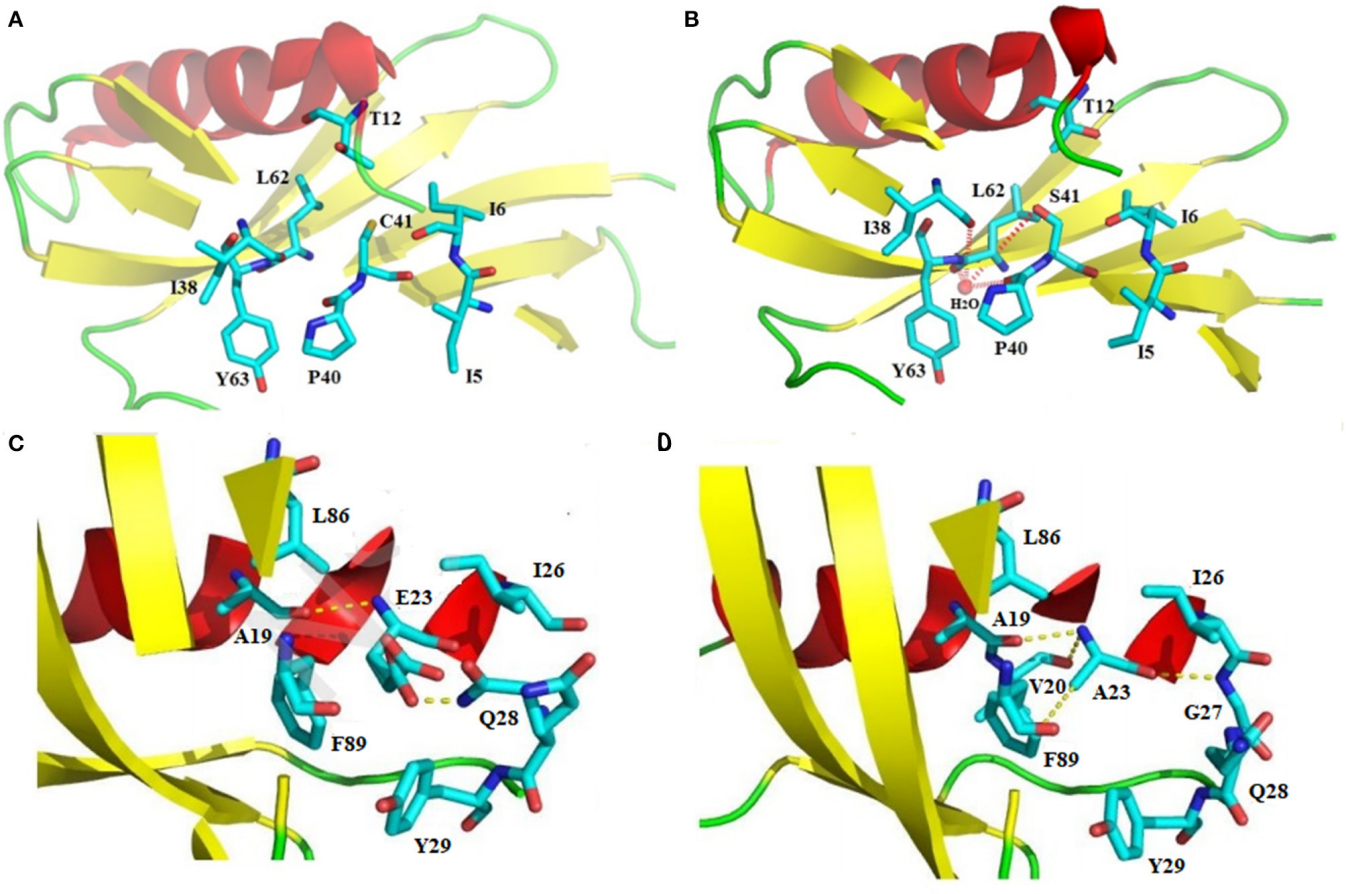
Structural illustration of the relationship between the intramolecular interaction patterns and the properties of sweet-tasting proteins. Formation of the intramolecular hydrogen bonds interaction network upon the C41S mutation of MNEI: **(A)** Spatial arrangement around C41 in the wild-type sweet-tasting protein monellin (PDB: 1IV7); **(B)** Formation of the intramolecular hydrogen bonds interaction network (red dashed lines) around the mutated S41, which accounts for the improved sweet potency of the mutants Q28K/C41S/Y65R and E23Q/Q28K/C41S/Y65R (PDB: 5LC6 and 5LC7). Comparison of the intramolecular interaction networks of the wild-type MNEI and its mutant E2N/E23A; **(C)** Intramolecualr interaction forces around the E23 site of the wild-type MNEI. The hydrogen bonds were indicated with yellow dashed lines (PDB: 2O9U); **(D)** Modified intramolecualr interaction network around the A23 site of E2N/E23A mutant (PDB: 5Z1P), which accounts for its significantly improved thermostability.

“Proteins sector” in sweet-tasting proteins has not been reported until now. By analyzing previous experimental data, we highlight herein that the intramolecular interaction forces in sweet-tasting proteins can significantly affect the sweetness of these proteins. For instance, a G16A mutation located in the core of sweet protein monellin could induce flexibility changes of protein surface via propagation effects mediated by hydrophobic interactions, which led to a 10-fold decrease of sweetness (42). Two mutants of MNEI Q28K/C41S/Y65R and E23Q/Q28K/C41S/Y65R were reported with around 1.5 to 2.5-fold enhancement of sweetness than the wild-type protein. We compared their solved structures (PDB: 5LC6 and 5LC7) with that of the wild-type (PDB: 1IV7) (43), and indicated that the mutated residue S41 in both mutants adopt different conformations relative to C41 in wild-type, which lead to formation of a hydrogen bond with a water molecule that connects mutated S41 to main chain atoms of P40, I38, and Y63 via hydrogen bonds (Figures 2A,B). Similar arrangement was also found for mutant Y65R that resulted in modified interactions in the protein (2). Therefore, it can be suggested that reorganizations of intramolecular interaction network (“protein sectors”) account for the conformations changes of the proteins and their orientation on the receptor, thus affecting their sweetness.

It was reported that the sweetness of a series of variants of the sweet-tasting protein brazzein is correlated to the patterns of hydrogen bonds detected by NMR spectroscopy. Specifically, three sweeter variants exhibited the same pattern of hydrogen bonds, whereas all three “non-sweet” variants lacked one hydrogen bond at the middle of the α-helix, where it is kinked, and one hydrogen bond in the middle of β-helixs II and III, where they are twisted (44). Similar structural rearrangements were also described in other variants of brazzein (45). These results highlight the significance of intramolecular interaction patterns (“protein sectors”) for the sweetness of sweet-tasting proteins.

Besides these findings, studies on multiple mutations indicated that there were combinatorial effects of mutated residues for the sweetness of sweet-tasting proteins. For example, the multiple-sites mutant H31R/E36D/E41A of sweet-tasting protein brazzein displayed significantly improved sweetness than those of three double-sites mutants (H31R/E36D, H31R/E41A, and E36D/E41A) and three single-site mutants (H31R, E36D, and E41A) (46). These results further underline the essential roles of intramolecular interaction organization (“protein sectors”) in the sweet-tasting proteins for their performance.

## “Proteins Sectors” Determines the Stability of Sweet-Tasting Proteins

Stability is another important property of sweet-tasting proteins, and their intramolecular interaction forces are shown to be also critical for their stability. For example, the most thermostable sweet protein brazzein harbors four intramolecular disulfide bonds, which are essential for its thermostability (47). Moreover, intramolecular disulfide bonds are prevalent in other sweet-tasting proteins, such as thaumatin and mabinlin (11, 14). We have solved the crystal structure of E2N/E23A mutant of MNEI (the single-chain monellin) (PDB: 5Z1P), and indicated that compared to the wild-type protein, mutation of E23 to A could resulted in new hydrogen bonds with V20 and G27 as well as an enhanced C-H…π bond interaction with F89, which are responsible for its improved thermostability (Tm values 84.9 and 74.2°C for E2N/E23A and wild-type MNEI, respectively) (Figures 2C,D) (48).

In another mutant of MNEI (E23Q/Q28K/C41S/Y65R), E23Q mutation was reported to induce conformational arrangements of surrounding residues and establish new hydrogen bonds with Y29 and G30. The Q28K mutation plays a concerted role, which hydrogen bonds with N90. All these new interactions establish a stabilizing hydrogen bonds network that account for the improved stability of the mutated protein (43). These results together suggest the crucial roles of intramolecular interaction patterns (“protein sectors”) for the stability of sweet-tasting proteins.

## Discussion and Future Outlook

Based on the above analysis, it is evident that “protein sector” in sweet-tasting proteins is significant for understanding their structure and function relationship, which is essential for the protein engineering of these biomacromolecules. However, our knowledge about the intramolecular interaction organization of sweet-tasting proteins, especially those determining their properties, is still limited. For instance, how connection patterns of each amino acid shape the full landscape of “protein sector” in the proteins and determine their properties? Is there an universal pattern among different sweet-tasting proteins (49)?

Because “protein sector” in sweet-tasting proteins is a global network composed by many different amino acids, thus in the future a large number of multiple mutations are needed to elucidate the function of each residue and their overall performances. Furthermore, because most findings related to the “protein sector” in sweet-tasting proteins are from monellin (or single chain monellin, MNEI), it is needed to perform more extensive studies toward other sweet-tasting proteins to illuminate the universal mechanism of intramolecular organization in these miraculous proteins. Moreover, more details of dynamic conformations of sweet-tasting proteins and their variants are promising to uncover the intrinsic assembling of “protein sectors” as well as their relationship with the properties of these proteins (50, 51). Prospectively, our new insight into the structure-activity relationship of sweet-tasting proteins-“protein sectors” would provide meaningful guidelines for their protein engineering, which could greatly accelerate the improvement of their properties and promote the application of sweet-tasting proteins in foods and beverages (52, 53).

## Data Availability Statement

The original contributions presented in the study are included in the article/supplementary material, further inquiries can be directed to the corresponding author/s.

## Author Contributions

BL and XZ conceived and designed the data analysis. CW and BL wrote the manuscript. XZ and YZ helped to analyze the data. All authors agree to be accountable for the content of this work.

## Conflict of Interest

YZ was employed by the company Shandong Aojing Biotechnology Co., Ltd., Zoucheng, China. The remaining authors declare that the research was conducted in the absence of any commercial or financial relationships that could be construed as a potential conflict of interest.
